# Defining the landscape of circRNAs in non-small cell lung cancer and their potential as liquid biopsy biomarkers: a complete review including current methods

**DOI:** 10.20517/evcna.2020.07

**Published:** 2021-06-06

**Authors:** Carlos Pedraz-Valdunciel, Rafael Rosell

**Affiliations:** ^1^Cancer Biology and Precision Medicine Department, Germans Trias i Pujol Research Institute and Hospital, Badalona 08916, Spain.; ^2^Biochemistry, Molecular Biology and Biomedicine Department, Universitat Autónoma de Barcelona, Bellaterra, Barcelona 08193, Spain.; ^3^Universitat Autónoma de Barcelona, Bellaterra, Barcelona 08193, Spain.

**Keywords:** CircRNA, extracellular vesicles lung cancer, NSCLC, liquid biopsies, biomarkers

## Abstract

Despite the significant decrease in population-level mortality of lung cancer patients as reflected in the Surveillance Epidemiology and End Results program national database, lung cancer, with non-small cell lung cancer (NSCLC) in the lead, continues to be the most commonly diagnosed cancer and foremost cause of cancer-related death worldwide, primarily due to late-stage diagnosis and ineffective treatment regimens. Although innovative single therapies and their combinations are constantly being tested in clinical trials, the five-year survival rate of late-stage lung cancer remains only 5% (Cancer Research, UK). Henceforth, investigation in the early diagnosis of lung cancer and prediction of treatment response is critical for improving the overall survival of these patients. Circular RNAs (circRNAs) are a re-discovered type of RNAs featuring stable structure and high tissue-specific expression. Evidence has revealed that aberrant circRNA expression plays an important role in carcinogenesis and tumor progression. Further investigation is warranted to assess the value of EV- and platelet-derived circRNAs as liquid biopsy-based readouts for lung cancer detection. This review discusses the origin and biology of circRNAs, and analyzes their present landscape in NSCLC, focusing on liquid biopsies to illustrate the different methodological trends currently available in research. The possible limitations that could be holding back the clinical implementation of circRNAs are also analyzed.

## INTRODUCTION

Lung cancer is the most commonly diagnosed cancer, contributing greatly to cancer incidence and cancer-related deaths worldwide^[1]^. Of those lung cancers, non-small cell lung cancer (NSCLC) accounts for 85% of the cases; the development of the disease is attributed to multileveled and elusive complex interactions between genetic liabilities, sex, environmental toxins, and imbalanced signaling processes.

Although the mortality rate of NSCLC has decreased in previous years, presumably due to the approval and routinization of targeted therapies and immunotherapies^[2]^, the prognosis in late-stage lung cancer remains dismal. While the 5-year overall survival (OS) of early-stage lung cancer is 85% (stage IA), these numbers fall to only 5% in late-stage cases (stage IV). In addition to tumor tissue characterization, liquid biopsies have been introduced to overcome, or complement, invasive tissue biopsies.

Not only are they instrumental in achieving early detection of the tumor, but they can also be exploited to monitor therapy resistance and provide a more heterogeneous readout of the tumor burden^[3]^. This allows the identification of resistance mechanisms and can guide second-line therapy selection.

Different body fluids can be used as liquid biopsies, including blood, urine, and saliva. Circulating molecules, such as cell-free DNA (cfDNA), RNA, or proteins, can either be freely present within these media or can be extracted and analyzed from circulating extracellular vesicles (EVs) or tumor-educated platelets (TEPs)^[4]^.

Lung cancer involves massive changes in RNA metabolism, both in the tumor and circulating EVs and TEPs. Traditional RNA biomarker discovery research for either lung cancer detection or monitoring of treatment response has mainly focused on the expression of mRNA and miRNA^[5-7]^.

Circular RNAs (circRNAs) are a recently re-discovered type of RNA generated by coupling the 5' and 3' ends in a non-canonical process known as back-splicing^[8]^. This circular structure lacks a poly(A) tail, making most of them resistant to the exonuclease RNase R and, therefore, making them robustly stable molecules compared to lineal mRNA. While thousands of circRNAs have been described thanks to the technological burst of deep sequencing^[9]^, only the function of a fraction has been elucidated.

Recent investigations have unveiled the role of circRNAs as important players in NCLSC, positioning them as valuable biomarkers for early detection and promising candidates for seeking therapeutic and prevention strategies^[10]^.

This review analyzes the current state of circRNA research, starting from their biology to their different functions and implications in NSCLC, with a special focus on their not yet fully exploited potential as liquid biopsy biomarkers. We also review the most recently discovered circRNAs, both in solid and liquid specimens.

In addition, we provide a practical and complete guide on the current methodology available for their study, stressing the current limitations that may be preventing their implementation in the clinical setting.

## CIRCULAR RNA EXPRESSION IN HUMANS

Although circRNAs have been acknowledged for many years as abnormally spliced “scrambled” transcripts^[11]^, only recently have they been re-defined as biologically active molecules with a significant role in human homeostasis, having a tissue-specific expression profile during the different stages of development^[12]^.

More than 60% of human genes can express circRNAs^[13]^. However, their expression levels in tissue remain rather low, accounting for only 5%-10% of the canonical (linear) mRNA expression^[14,15]^.

CircRNAs are originated by an alternative process called “back-splicing”, where the 5' splice donor can stick to the 3' splice acceptor of an upstream exon. This process results in forming a circular structure that can include one or different exonic/intronic regions, depending on the specific mechanism that was inferred during this non-canonical process^[16]^.

**Figure 1:**
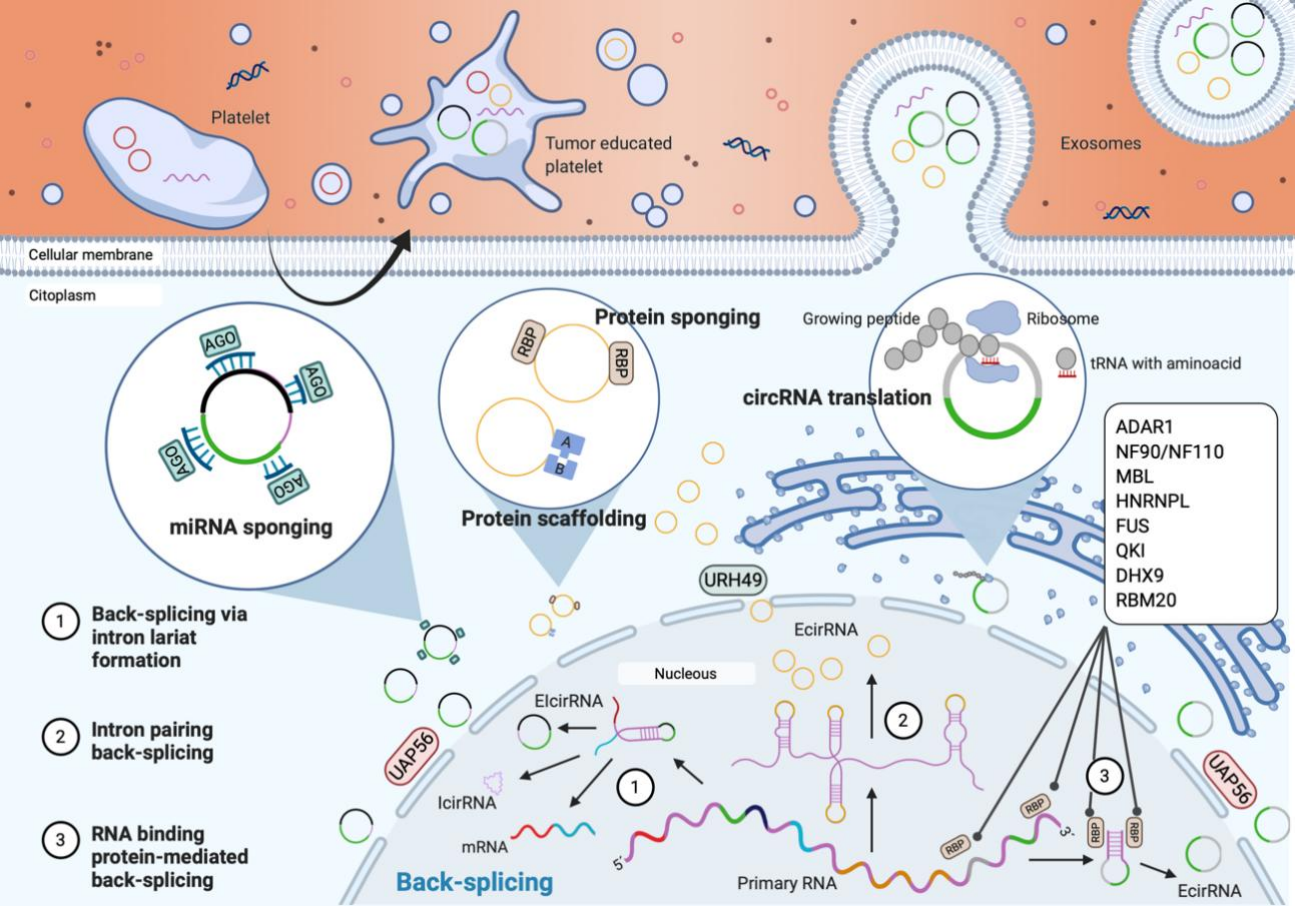
Biosynthesis and molecular functions of circRNAs. CircRNAs are generated by three different mechanisms of back-splicing (via lariat formation, intron pairing or RNA binding proteins). Resultant circRNAs can be formed by only exonic regions (EcircRNAs), intronic regions (IcircRNAs) or both (EIcircRNAs). circRNAs are exported into the cytoplasm in a size-mediated manner by URH49 and UAP56. Once in the cytoplasm, circRNAs will perform their functions including miRNA and protein sponging, protein scaffolding, or even translate into small functional peptides. CircRNAs will be released into the blood stream inside exosomes mediating cellular communication. Most cellular types, including tumor cells, will secrete circRNA-containing EVs. Platelets can modify its content when in contact with the tumor, including their circRNA expression profile.

They have arisen as key post-transcriptional regulators throughout different functions [Figure 1], with micro-RNA (miRNA) sponging being the most studied. During this process, the circRNA binds to the argonaute-miRNA complex, and either via miRNA degradation or inhibition of the miRNA-mRNA interaction, it triggers further mRNA expression^[17]^.

Recent studies have also revealed that circRNAs could associate with ribosomes and be translated into functional short peptides, in a cap-independent manner^[18]^. Alternatively, they can also associate with proteins acting as scaffolding for enzymatic reactions. The process of circRNA synthesis generates an imbalance of the canonical splicing; hence, the back-splicing process itself stands as a direct regulator of the circRNA precursor gene at the transcriptional level.

### Biosynthesis and regulation of circRNAs

Different back-splicing mechanisms have been reported in the nucleus, including RNA binding protein (RBP)-mediated circularization, circRNA synthesis by intron pairing, or circularization by intron-lariat formation^[16] ^[Figure 1]. The first mechanism is normally executed by associating two adjacent exons and skipping the intronic region during an RBP-assisted circularization process, resulting in an exonic-circRNA (EcircRNA). Numerous RBPs have been described to regulate this mechanism, such is the case of the adenosine deaminase RNA specific-1 protein (ADAR1)^[19]^, NF90/NF110 immune factors^[20]^, muscleblind transcription factor (MBL)^[21]^, heterogeneous nuclear ribonucleoprotein L^[22]^, FUS protein^[23]^, Quaking binding protein (QKI)^[24]^, RNA helicase DHX9^[25]^, and the RNA-binding motif protein 20^[26]^.

Exon-intron circRNAs are the result of 2 or more exons circularized along with their corresponding introns via intron-lariat formation. Intron pairing back-splicing is usually the common process in conserved RNAs with high frequency of *Alu* repeats in flanking sequences. These *Alu* elements complement each other, promoting the hairpin formation and further back-splicing, creating mono-EcircRNAs as a result^[27]^. Intronic circRNAs are another type of such a class; however, the mechanism of generation of these molecules remains yet unclear. 

After synthesis in the nucleus, circRNAs are exported into the cytoplasm. Recent studies have shown the active role of the UAP56/URH49 helicases in this size-mediated process. UAP55 is required to transfer molecules longer than 1300 nucleotides, while URH49 intervenes only in short transcript exporting^[28]^. Once in the cytoplasm, circRNAs accumulate and exert their function by regulating transcription, normally via sponging targeted miRNAs.

How circRNA gets degraded still remains unclear; however, recent investigation has shed light on this conundrum, unveiling some intriguing mechanisms that underpin circRNA decay. Hansen *et al*.^[29]^ describe an Ago2-miR-671-mediated degradation of the circRNA CDR1as (aka ciRS-7). In another study by Park *et al*.^[30]^, a cleavage mechanism induced by RNase P/MRP was elucidated in N6-methyladenosine (m6A)-enriched circRNAs. More recently, a study by Liu *et al*.^[31]^ demonstrated that some circRNAs tend to form intricate duplexes which makes them susceptible to degradation by RNase L upon viral infection.

A different mechanism was described by Fischer *et al*.^[32]^ revealing an alternative structure-mediated circRNA regulation process that selectively degrades circRNAs based on 3'-UTR structure complexity via the UPF1/G3BP1 protein complex.

## CIRCULAR RNAS IN NSCLC

The implication of circRNAs in cancer metabolism has been studied in recent years. Their contribution to mutant glycolysis (via transporter, enzyme, and/or transcription factor regulation), lipogenesis and lipolysis, glutaminolysis, and oxidative respiration has been widely demonstrated^[33]^.

CircRNAs are becoming a new area of interest within cancer research, including NSCLC, where several authors are contributing by investigating the effect that dysregulated circRNA expression can have on the different cancer stages. Although their implication in NSCLC has not been as intensively investigated as other types of non-coding RNAs, circRNAs have been shown to have a significant role in tumorigenesis, tumor development, proliferation, migration, invasion, and sensitivity to NSCLC therapy^[34]^. In light of these aforementioned findings, recent publications highlight the potential of these circular transcripts as plausible biomarkers to assess disease status.

### CircRNAs as biomarkers of NSCLC

The number of studies on circRNA profiling in NSCLC patients has exploded exponentially in the last few years [Table 1].

**Table 1:** List of the most relevant recently discovered circRNAs associated with lung cancer

**circRNA**	**Gene**	**CircBase ID**	**Source**	**Regulation**	**Target**	**Downstream pathway**
circFGFR3	*FGFR3*	-	NSCLC tissues	Upregulated	hsa-miR‐22‐3p	Galectin‐1‐AKT/ERK1/2
ircNOL10	*NOL10*	hsa_circ_0000977	LC cells	Downregulated	hsa-miR-7	SCML1
ciRS-7	*CDR1*	-	NSCLC tissues and cell lines	Upregulated	-	-
circABCC4	*ABCC4*	hsa_circ_0030586	LUAC tissues and cell lines	Upregulated	hsa-miR‐3186‐3p	TNRC6B axis
circCDR1	*CDR1*	hsa_circ_0001946	LUAC tissues and cell lines	Upregulated	hsa-miR-135a-5p	SIRT1/Wnt/β-catenin
circATXN7	*ATXN7*	hsa_circ_0007761	LC tissues and cell lines	Upregulated	-	-
circATAD3B	*ATAD3B*	hsa_circ_0000003	NSCLC tissues and cell lines	Upregulated	hsa-miR-338-3p	IRS2
circP2RX1	*P2RX1*	hsa_circ_0000735	NSCLC tissues and cell lines	Upregulated	hsa-miR-1179, miR-1182	-
circC16orf62	*C16orf62*	hsa_circ_0003645	NSCLC tissues and cell lines	Upregulated	hsa-miR-1179	TMEM14A
circPDZD8	*PDZD8*	hsa_circ_0020123	NSCLC tissues and cell lines	Upregulated	hsa-miR-488e3p	ADAM9
circTUBA1C	*TUBA1C*	hsa_circ_0026134	NSCLC tissues and cell lines	Upregulated	hsa-miR-1256, miR-12	TCTN1 and GAGE1
circCAMK2A	*CAMK2A*	hsa_circ_0128332	LUAD	Upregulated	hsa-miR-615-5p	Fibronectin 1
circFOXM1	*FOXM1*	hsa_circ_0025033)	NSCLC tissues and cell lines	Upregulated	hsa-miR-1304-5p	PPDPF and MACC1
circMTO1	*MTO1*	hsa_circ_0007874	LUAD tissues and cell lines	Downregulated	hsa-miR-17	QKI-5
circPRMT5	*PRMT5*	hsa_circ_0031250	NSCLC tissues and cell lines	Upregulated	hsa-miR-377/382/498	EZH2
circRAD23B	*RAD23B*	hsa_circ_0087855	NSCLC tissues and cell lines	Upregulated	hsa-miR-593e3p, hsa-miR-653e5p	CCND2 and TIAM1
circZKSCAN1	*ZKSCAN1*	hsa_circ_0001727	NSCLC tissues and cell lines	Upregulated	hsa-miR-330-5p	FAM83A (MAP signaling)
circCRIM1	*CRIM1*	hsa_circ_0002346	LUAC cell lines	Downregulated	hsa-miR‐182/miR‐93	-
circHIPK3	*HIPK3*	hsa_circ_0000284	A549, H838 cell lines	Upregulated	hsa-miR-124-3p, miR-149	STAT3-PRKAA/AMPKα
circPDK1	*PDK1*	hsa_circ_0006006	LUSC tissues	Upregulated	-	-
circPIP5K1A	*PIP5K1A*	hsa_circ_0014130	NSCLC cell lines	Upregulated	hsa-miR‐600	HIF-1α
circPRKCI	*PRKCI*	hsa_circ_0067934	NSCLC cell lines	Upregulated	hsa-miR-545, hsa-miR-589	E2F7
circPTPRA	*PTPRA*	hsa_circRNA_0102984	NSCLC tissues and cell lines	Downregulated	hsa-miR-96-5p	RASSF8/E-cadherin
circPVT1	*PVT1*	Hsa_circ_0001821	NSCLC tissues and cell lines	Upregulated	hsa-miR-497	-
circTP63	*TP63*	hsa_circ_0068515	LUSC tissues and cell lines	Upregulated	hsa-miR-873-3p	FOXM1/CENPA-CENPB
circVANGL1	*VANGL1*	-	NSCLC tissues and cell lines	Upregulated	hsa-miR-195	Bcl-2
circZFR	*ZFR*	hsa_circ_0001649	NSCLC tissues and cell lines	Upregulated	hsa-miR-101-3p	CUL4B
circMras	*MRAS*	hsa_circ_0067512	LUAC samples and NSCLC cell lines	Downregulated	hsa-miR‐567	PTPRG
F-circSR	*SLC34A2-ROS1*	-	HCC78 cell line	Upregulated	-	ROS
circCDK6	*CDK6*	hsa_circ_000984	NSCLC tissues and cell lines	Upregulated	-	Wnt/β-catenin pathway
circRUNX1	*RUNX1*	hsa_circ_0002360	LUAC tissues	Upregulated	hsa-mir-3620-5p	PHF19
circZNF720	*ZNF720*	hsa_circ_0007059	LC tissues and cell lines	Downregulated	hsa-miR-378	Wnt/β-catenin and ERK1/2
circRNF121	*RNF121*	hsa_circ_0023404	NSCLC tissues and cell lines	Upregulated	hsa-miR-217	ZEB1
circTADA2A	*TADA2A*	hsa_circ_0043278	NSCLC tissues and cell lines	Upregulated	hsa-miR-520f	ROCK1, CDKN1B and AKT3
circLIFR	*LIFR*	hsa_circ_0072309	NSCLC tissues and cell lines	Downregulated	hsa-miR-580-3p	-
circITCH	*ITCH*	N.A.	LC tissues and cell lines	Downregulated	hsa-miR-7 and hsa-miR-214	(PI3K)/AKT
circSMARCA5	*SMARCA5*	hsa_circ_0001445	NSCLC tissues and cell lines	Downregulated	hsa-miR-19b-3p	HOXA9
circRAD23B	*RAD23B*	hsa_circ_0087862	NSCLC tissues and cell lines	Upregulated	hsa-miR-1253	RAB3D
circPIP5K1A	*PIP5K1A*	hsa_circ_0014130	NSCLC tissues and cell lines	Upregulated	hsa-miR-142-5p, hsa-miR-136-5p	IGF-1 and BCL2
circABCB10	*ABCB10*	hsa_circ_0008717	NSCLC tissues and cell lines	Upregulated	-	KISS1
circIGF1R	*IGF1R*	hsa_circ_0005035	NSCLC tissues and cell lines	Downregulated	hsa-miR-1270	VANGL2
circSOX4	*SOX4*	N.A.	LUAD tissues and cell lines	Upregulated	hsa-miR‑1270	PLAGL2 (WNT signaling)
circACACA	*ACACA*	hsa_circ_0043256	NSCLC tissues and cell lines	Upregulated	hsa-miR-1183	PI3K/PKB pathway
circBIRC6	*BIRC6*	hsa_circ_0003288	NSCLC tissues and cell lines	Upregulated	hsa-miR-145	FSCN1 and S6K1
circCCDC66	*CCDEC66*	N.A.	NSCLC cell lines	Upregulated	hsa-miR-33a-5p	KPNA4/STAT3
circGFRA1	*GFRA1*	hsa_circ_0005239	NSCLC tissues and cell lines	Upregulated	hsa-miR-188-3p	PI3K/AKT
circLARP4	*LARP4*	N.A.	NSCLC tissues and cell lines	Downregulation	-	SMAD7
circTCONS	*TCONS*	hsa_circ_0000326	NSCLC tissues and cell lines	Upregulated	hsa-miR-338-3p	RAB14
circDHCR24	*DHCR24*	hsa_circ_0012673	LC tissues and cells	Upregulated	hsa-miR-320a	LIMK18521
circMACF1	*MACF1*	hsa_circ_0011780	NSCLC tissues and cells	Downregulated	hsa-miR-544a	FBXW7
circPANX2	*PANX2*	hsa_circ_0012515	NSCLC tissues and cells	Upregulated	hsa-miR-98-5p, hsa-miR-615-5p, hsa-let-7a-5p, hsa-let-7b-5p and hsa-let-7c-5p	-
circMET	*MET*	hsa_circ_0082003	NSCLC tissues and cells	Upregulated	miR-145-5p	CXCL3

ciRS-7 was the first and best characterized circRNA in cancer and served as a foundation stone for current research. Its role in carcinogenesis was first described in hepatocellular carcinoma, following breast and cervical cancer, acting as a competing endogenous RNA for miR-7^[35]^. A recent study has introduced ciRS-7 as an important player in lung cancer; its expression seems to correlate with tumor size and both lymph and tumor node metastasis stages^[36]^.

A study by Wang *et al*.^[37]^ recently demonstrated the involvement of circSOX4 in lung adenocarcinoma by activating the WNT signaling pathway via sponging miR-1270 and following upregulation of PLAL2. CircSOX4 was found overexpressed in all managed lung adenocarcinoma tissue samples, and further validated across different cell-based preclinical experiments^[37]^.

Circular RNA HIPK3 (circHIPK3) is yet another extensively studied circRNA critical in cell proliferation of different types of cancer^[38]^. Its specific role in NSCLC has been recently discovered by Xie *et al*.^[39]^ demonstrating impaired cell proliferation, migration, invasion and autophagy induction via the miR124-3p-STAT3-PRKAA/AMPKa axis upon silencing of the cited circular transcript. Authors also demonstrated that overexpression of circHIPK3 correlates to poor survival, especially in advanced stages.

Another well studied circRNA, circSMARCA5, plays a significant role in NSCLC via the miR-19b-3p/HOXA9 axis, setting the grounds for exploring underlying therapeutic targets^[40]^. On a similar note, a circular RNA from FGFR3 was reported in NSCLC, promoting cell invasion and proliferation of tumors by sequestering miR-22-3p, thus promoting galectin‐1, p‐AKT, and p‐ERK1/2 expression, and activating downstream pathways^[41]^.

The oncogenic circ-FOXM1 was first discovered overexpressed in pancreatic tissues upregulating the pancreatic progenitor cell differentiation and proliferation factor (PPDPF) and metastasis-associated in colon cancer 1 (MACC1) proteins via miR-1304-5p sponging. More recently, the same circ-FOXM1/miR-1304-5p/PPDPF/MACC1 axis was found decisive for NSCLC development and progression^[42]^.

Chromosomal translocations are cancer-associated events that may strike frequently in some genes, like *ROS* or *ALK*, leading to activation of downstream signaling pathways upon sustained expression^[43]^. These events can also generate oncogenic circRNAs, as has been reported with the solute carrier family 34 member 2 (SLC34A2) and ROS proto-oncogene 1 (ROS1), producing two circRNAs (F-circSR1 and F-circSR2) both promoting cell migration in NSCLC^[44]^.

Precursor mRNA of driver mutations, such as MET, can also lead to the generation of circRNAs. CircMET was first described in hepatocellular carcinoma driving immunosuppression and anti-programmed cell death 1 (PD-1) therapy resistance via the miR-30-5p/snail/DPP4 axis^[45]^. Its role in NSCLC was recently discovered promoting tumor proliferation via the miR-145-5p/CXCL3 axis^[46]^.

Although a circRNA from epidermal growth factor receptor (EGFR) has been reported in mouse ovaries during postnatal development with a marked expression profile, the implication of this circRNA in lung cancer has not been studied yet.

There have been no circRNAs derived from the *KRAS* gene reported either; however, numerous circRNAs have been portrayed as key intermediaries of the classical pathways and may serve as a readout of these foremost altered genes.

### CircRNAs as biomarkers of treatment resistance in NSCLC

Although several studies have unveiled the potential role of circRNAs in lung cancer development and progression, not much has been clarified regarding their contribution to therapeutic resistance, and only a few published studies focus on their involvement in this area [Table 2]. CircRNAs can be classified as promoters, when their high expression enhances resistance to cancer therapy; or suppressors, when their expression limits the progression of the disease during treatment, thus acting as inhibitors of resistance.

**Table 2:** List of circRNAs involved in NSCLC treatment resistance

**circRNA**	**Gene**	**CircBase ID**	**Source**	**Regulation**	**Resistance**	**Drug**	**Target**	**Downstream pathway**	**Ref.**
circSEMA5A	*SEMA5A*	hsa_circ_0071799	NSCLC cells	Upregulated	Chemotherapy	Taxol	hsa-miR-141-5p; also, hsa-miR-1228-5p, hsa-miR-194-3p, hsa-miR-512-5p, hsa-miR-4-5p	-	Xu *et al*.^[50]^, 2018
circFLNA	*FLNA*	hsa_circ_0091931	NSCLC cells	Downregulated	Chemotherapy	Taxol	hsa-miR-34c-5p; also, hsa-miR-105-3p, hsa-miR-1268b, hsa-miR-1226-5p, hsa-miR-1180	-	Xu *et al*.^[50]^, 2018
circMTDH.4	*SNORD115*	-	NSCLC tissue and cell lines	Upregulated	Chemotherapy	5‐FU, cisplatin	hsa-miR-630	AEG‐1	Li *et al*.^[47]^, 2020
circESRP1	*ESRP1*	hsa_circ_0084927	Lung cancer cells	Downregulated	Chemotherapy	Generic chemotherapy	hsa-miR-93-5p	TGF-β pathway	Huang *et al*.^[55]^, 2020
circARFGEF2	*ARFGEF2*	hsa_circ_0003998	LUAC cells	Upregulated	Chemotherapy	Docetaxel	hsa-miR-326	-	Yu^[51]^, 2019
circCDR1	*CDR1*	hsa_circ_0001946	A549 cell line	Downregulated	Chemotherapy	Cisplatin	hsa-miR-7-5p, hsa-miR-671-5p, hsa-miR-1270, hsa-miR-3156-5p	NER signaling	Huang *et al*.^[52]^, 2019
circPGC	*PGC*	hsa_circ_0076305	NSCLC tissues and cell lines	Upregulated	Chemotherapy	DDP	hsa-miR-296-5p	STAT3	Dong *et al*.^[48]^, 2019
circAKT3	*AKT3*	hsa_circ_0017252	Lung cancer tissues and cell lines	Upregulated	Chemotherapy	DDP, cisplatin	hsa-miR-516b-5p	STAT3	Xu *et al*.^[49]^, 2020
circPVT1	*PVT1*	hsa_circ_0001821	LUAC tissues and cell lines	Upregulated	Chemotherapy	Cisplatin, pemetrexed	hsa-miR-145-5p	ABCC1	Zheng *et al*.^[53]^, 2020
circCDK14	*CDK14*	hsa_circ_0004015	NSCLC cells	Upregulated	Tyrosine Kinase Inhibitors (TKIs)	Gefitinib	hsa-miR-1183	PDPK1 gene	Zhou *et al*.^[56]^, 2019
circKRT17	*KRT17*	hsa_circ_0043632	AZD9291-resistant NSCLCcell lines	Upregulated	Tyrosine Kinase Inhibitors (TKIs)	Osimertinib	hsa-miR-6861-3p, hsa-miR-492, hsa-miR-4743-5p, hsa-miR-6829-3p, hsa-miR-6778-3p	-	Chen *et al*.^[58]^, 2019
circFXYD3	*FXYD3*	hsa_circ_0050581	AZD9291-resistant NSCLCcell lines	Downregulated	Tyrosine Kinase Inhibitors (TKIs)	Osimertinib	hsa-miR-6722-5p, hsa-miR-4641, hsa-miR-4707-3p, hsa-miR-4258, hsa-miR-652-3p	-	Chen *et al*.^[58]^, 2019
circFGFR1	*FGFR1*	hsa_circ_0084003	NSCLC tissues and cells	Upregulated	Immunotherapy	Anti-PD-1 therapy	hsa-miR-381-3p	PD-1	Zhang *et al*.^[59]^, 2019

Astrocyte elevated gene-1 (AEG-1) is a key player in development, progression, and metastasis of lung cancer by regulating the Wnt/β-catenin pathway. In a recent publication, Li *et al*.^[47]^ showed that circMTDH.4 regulates AEG-1 expression by sponging miR-630, leading to chemo- and radio-resistance in NSCLC cells. Sensitivity was restored via the knockdown of the cited circRNA or over expression of its target, miR-630.

Two different works have recently been published describing circRNAs that regulate the expression of STAT3. Dong *et al*.^[48]^ reported that upregulation of hsa_circ_0076305 confers DDP-resistance to NSCLC cells via sponging miR-296-5p, positively modulating STAT3. Xu *et al*.^[49]^ introduced the role of circAKT3 inhibiting cisplatin sensitivity by regulating mir-516b-5p/STAT3 axis.

Other important circRNAs described to be involved in chemotherapy resistance are hsa_circ_0071799 via miR-141 (taxol resistance)^[50]^, hsa_circ 0091931 via miR-34c-5p^[10]^, hsa_circ_0003998 via miR-326^[51]^, hsa_circ_0001946 via miR-7-5p, miR-671-5p, miR-1270 and miR-3156-5p (NER signaling, cisplatin resistance)^[52]^, circPVT1 via miR-145-5p (ABCC1, cisplatin, and pemetrexed resistance)^[53]^, circNFIX via miR-132 (TMZ-resistant)^[54]^, and cESRP1. Huang *et al*.^[55]^ recently discovered a suppressor circRNA that, when downregulated, allows major expression of its target miR-93-5p. This process leads to the upregulation of downstream targets, such as Smad7/p21(CDKN1A), enhancing the transforming growth factor-β (TGF-β) pathway. Furthermore, cESRP1 overexpression boosts cisplatin sensitivity by repressing miR-93-5p and TGF-β pathway in SCLC. Related to this pathway, PDPK1, intermediary of the PI3K/AKT/mTOR pathway, has been discovered to be regulated by the hsa_circ_0004015-miR-1183 axis^[56]^. Overexpression of this circRNA can induce gefitinib resistance in NSCLC cells by sponging the abovementioned miRNA.

Other authors have centered their investigation on the differential expression of circRNAs that confer resistance to this and other tyrosine kinase inhibitor-based therapies. Fu *et al*.^[57]^ found hsa_circRNA_012515 increased in gefitinib-resistant NSCLC cell lines. Further investigation in patient tissue indicated that high expression correlated with lower OS and shorter progression free survival. Chen *et al*.^[58]^ found 10 differentially expressed circRNAs in different osimertinib-resistant lung cancer cell lines. Five of them were further validated and proved to correlate with resistance status (hsa_circ_0043632, hsa_circ_0048856, hsa_circ_0043634, hsa_circ_0050581, and hsa_circ_0023302)^[58]^. The authors made use of specific software to predict possible targeted miRNAs; however, the axis or mechanism of action has not yet been elucidated.

CircRNAs seem to also have a role mediating response to immunotherapy. CircFGFR1 has been described by Zhang *et al*.^[59]^ to promote progression and anti-PD-1 resistance. By sponging miR-381-3p in NSCLC cells, C-X-C motif chemokine receptor 4 would result upregulated, leading to progression and resistance to therapy.

## CURRENT LANDSCAPE OF CIRCULAR RNAS IN LIQUID BIOPSIES AS NSCLC BIOMARKERS

Non-coding RNA-enriched exosomes are strategic players in different cancer stages, especially regarding malignant tumor metastasis^[60]^. The assessment of circRNA expression by RNAseq analysis in extracellular vesicles was first reported by Li *et al*.^[61]^, finding circRNAs enriched at least 2-fold in exosomes compared to producer cells. Although some authors defend the theory that exosomal circRNA enrichment may be a mechanism of cellular circRNA clearance^[62]^, few investigators have shown that these circRNA are directly involved in cellular communication, henceforth, acting as direct readouts of several human malignancies, including NSCLC^[63]^.

As a result, circRNAs stand as important liquid biopsy-derived biomarkers, holding potential for NSCLC diagnosis and prediction of treatment response^[64]^.

In a recent study, Chen *et al*.^[65]^ performed high throughput sequence of plasma-EV RNA cargo of lung adenocarcinoma patients, finding 182 circRNA dysregulated when compared to cancer-free donors, including 105 up-regulated and 78 downregulated. Four upregulated circRNAs were successfully validated by qRT-PCR (hsa_circ_0001492, hsa_circ_0001346, hsa_circ_0000690, and hsa_circ_0001439)^[65]^. Although authors elucidated the specific circRNA-miRNA-mRNA interaction, not much information about their biological impact was provided.

Fei *et al*.^[66]^ also presented in a recent study a novel circRNA, hsa_circRNA_005661, that could be found enriched in plasma EVs from lung adenocarcinoma patients with lymph node metastasis, presenting it as a biomarker of such stage^[66]^.

Not only plasma-EVs, but serum and whole plasma can serve as a good source of circRNAs [Table 3]. Xian *et al*.^[67]^ studied the circRNA differential expression profile in serum EVs from NSCLC patients. As a result, 3 circRNA stood out showing suitable biomarker potential (hsa_circ_0047921, hsa_circ_0007761, and hsa_circ_0056285) with the later correlating with clinical stages and lymph node metastasis in all Chinese patients included in the study^[67]^.

**Table 3:** List of the most relevant liquid biopsy-based circRNAs associated with NSCLC

**circRNA**	**Gene**	**circBase ID**	**Source**	**Expression**	**Target**	**Ref.**
circERBB2IP	*ERBB2IP*	hsa_circ_0001492	LUAD plasma exosomes	Upregulated	hsa-miR-130b-5p, hsa-miR-5195-3p, hsa-miR-4464, hsa-miR1236-3p, hsa-miR-106a-3p	Chen *et al*.^[65]^, 2019
circRNF13	*RNF13*	hsa_circ_0001346	LUAD plasma exosomes	Upregulated	hsa-miR-34B-5P, ha-miR-654-3p, hsa-miR-5683, hsa-miR-4452, hsa-miR-4662b	
circITGAL	*ITGAL*	hsa_circ_0000690	LUAD plasma exosomes	Upregulated	hsa-miR-7161-3p, hsa-miR-9-5p, hsa-miR-6843-3p, hsa-miR-4502, miR-372-5p	
circSCLT1	*SCLT1*	hsa_circ_0001439	LUAD plasma exosomes	Upregulated	hsa-miR-3671, hsa-miR-452-5p, hsa-miR-892c-3p, hsa-miR-223-3p, hsa-miR-4676-3p	
circCD226	*CD226*	hsa_circ_0047921	NSCLC serum exosomes	Downregulated	hsa-miR-let-7g	Xian *et al*.^[67]^, 2020
circATXN7	*ATXN7*	hsa_circ_0007761	NSCLC serum exosomes	Upregulated	-	
circRALB	*RALB*	hsa_circ_0056285	NSCLC serum exosomes	Downregulated	-	
circNPHP4	*NPHP4*	hsa_circ_0005661	LUAD plasma exosomes	Upregulated	-	He *et al*.^[66]^, 2020
circFARSA	*FARSA*	hsa_circ_0000896	NSCLC plasma	Upregulated	hsa-miR-330‐5p, hsa-miR-326, hsa-miR-1270	Hang *et al*.^[68]^, 2018
circCCCNB1	*CCCNB1*	hsa_circ_0001495	NSCLC plasma	Upregulated	-	
circVRK1	*VRK1*	hsa_circ_0000566	NSCLC plasma	Upregulated	-	
circCCDC134	*CCDC134*	hsa_circ_0001238	NSCLC plasma	Upregulated	-	
circZCCCJC6	*ZCCCJC6*	hsa_circ_0007037	NSCLC plasma	Upregulated	-	
circ_c1orf116	*C1ORF116*	hsa_circ_0141539	NSCLC plasma	Upregulated	-	
circPMS1	*PMS1*	hsa_circ_0001083	NSCLC plasma	Upregulated	-	
circDNA2	*DNA2*	hsa_circ_0006451	NSCLC plasma	Upregulated	-	
PcircSD3	*SD3*	hsa_circ_0004458	NSCLC plasma	Upregulated	-	
circSMAD2	*SMAD2*	hsa_circ_0000847	NSCLC plasma	Upregulated	-	
circYWHAZ	*YWHAZ*	hsa_circ_0005962	LUAD plasma	Upregulated	hsa-miR-369-5p, hsa-miR-626, hsa-miR-326, hsa-miR-330-5p, hsa-miR-1265, and hsa-miR-622	Liu *et al*.^[69]^, 2019
circBNC2	*BNC2*	hsa_circ_0086414	LUAD plasma	Downregulated	-	
F-circEA	*EMLK4-ALK*		Lung cancer tissues, plasma and cells	Upregulated	-	Tan *et al*.^[70]^, 2018
circZNF91	*ZNF91*	hsa_circ_0109320	NSCLC plasma	Downregulated	-	Liu *et al*.^[74]^, 2019
circZNF117	*ZNF117*	hsa_circ_0134501	NSCLC plasma	Upregulated	-	

Hang *et al*.^[68]^ explored the use of circRNA found in total plasma of NSCLC patients in order to find some candidates that could correlate to malignancy status. Not only did they find a notorious circRNA coming from the *FARSA* gene, *circFARSA*, but they also found a set of differentially expressed circRNAs (hsa_circ_0001495, hsa_circ_0000566, hsa_circ_0001238, hsa_circ_0007037, circ_c1orf116, hsa_circ_0001083, hsa_circ_0006451, hsa_circ_0004458, and hsa_circ_0000847) based on which they were able to discriminate NSCLC patients from healthy individuals. Additionally, they performed *in silico* investigation of possible targets of circFARSA. Consequently, miR‐330‐5p and miR‐326 emerged as direct target candidates. Both miR‐330‐5p and miR‐326 may interact directly with fatty acid synthase, which has been described as a notorious oncogene in various types of cancer^[68]^.

Also, directly from plasma Liu *et al*.^[69]^ found a two circRNA-based signature that could potentially be used to classify lung adenocarcinoma patients. Hsa_circ_0005962 was found upregulated while hsa_circ_0086414 was barely expressed. In addition, they observed that overexpression of hsa_circ_0005962 was correlated to mutant *EGFR* expression. *In vitro* experiments suggested that this circRNA could be involved in cancer proliferation.

Moreover, a fusion-gene *circRNA* has been studied in liquid biopsies. Tan *et al*.^[70]^ started their line of research exploring the existence of a circRNA derived from the fusion gene *EML4-ALK* (*F-circEA*) in the NCI-H2228 cell line. After verification, they observed that overexpression of this circRNA could trigger cell migration and invasion, contributing to tumor development. They validated the existence of this circRNA in plasma of NSCLC patients with the EML4-ALK translocation, suggesting that screening of plasma F-circEA in this type of patients could be a valuable approach to monitor the EML4-ALK translocation, and provide further guidance on targeted therapy.

Alhasan *et al*.^[71]^ showed for the first time that platelets are enriched in circRNAs when compared to nucleated tissues, and also, that their content is superior to that on mRNA. Preußer *et al*.^[72]^ demonstrated that platelets are not only a good source of circRNA, but also platelet-derived extracellular vesicles are enriched in these biomolecules, representing yet another source of potential biomarkers that may be involved in different signaling pathways.

Platelets change their RNA profile when in contact with the tumor, enabling them to contribute to the systemic and local responses to tumor growth. As a result, TEP-RNA can be used as a potential biomarker for cancer diagnostics^[73]^. Although TEPs could also possibly be enriched in circRNAs, and hold potential value for NSCLC diagnosis, nothing yet has been investigated.

Little has been elucidated regarding NSCLC treatment resistance based on liquid biopsy-based circRNAs. A study of Yu-Tao *et al*.^[74]^ comparing gefitinib responder and non-responder NSCLC patients found that higher expression of hsa_circ_0109320 in plasma correlated with longer progression free survival in gefitinib-treated NSCLC patients^[74]^; however, no information on the potentially affected signaling pathway has been provided.

### Current available methods for the study of circRNAs in liquid biopsies

Although there are different methods currently available for the study of circRNAs [Table 4], no consensus has been reached on which protocol to follow for either tissue or liquid biopsy-based circRNA expression analysis.

**Table 4:** Current methods for circRNA study

**Method**	**Application**	**Total RNA input**	**Advantages**	**Disadvantages**	**Ref.**
RNAseq	circRNA discovery	Normally ≥ 1µg is needed; however, 1 ng has been used in liquid biopsies showing good results	- Allows whole transcriptome sequence analysis, including rare and low abundant circRNAs	- Time consuming - It involves high quality RNA - Requires expertise for library preparation, sequencing, and Bioinformatics, for data normalization and analysis	Cheng *et al*. ^[125]^
Microarrays	circRNA discovery	2 µg	- Highly sensitive and specific for circRNA profiling - Easy technology, commercial arrays ready to use	- Although it may be possible to work with less RNA, recommended input remains rather high - circRNA discovery gets restricted to the amount of circRNA included in the panel - Requires Bioinformatics expertise for data normalization and analysis	Valladares-Ayerbes *et al*. ^[126]^
nCounter	circRNA discovery and quantification	85 ng	- Allows multiplexed analysis of up to 800 circRNA targets - Does not require amplification (if enough RNA input) - Works well with low quality RNA samples - Very little hands-on time, with results ready within 24 h - User-friendly data analysis software reducing the need for Bioinformatics support	- circRNA discovery gets restricted to the amount of circRNA included in the panel - Technology is costly, and constrained by one company	Zhang *et al*.^[127]^ Dahl *et al*.^[85]^, 2018
qRT-PCR	circRNA quantification	250 ng (3 replicas, 1 gene)	- Well-established technology - Cost-effective	- Does not allow analysis of a large number of genes - Susceptible to template switching and rolling circle amplification bias	
SplintQuant	circRNA quantification	2 nM	- Sensitive and specific approach - Highly reproducibility rates - Eludes the template switching and rolling circle amplification bias found with qRT-PCR	- Novel protocol - No tested in liquid biopsies	Conn^[92]^, 2019
RT-PCR + end-point PCR + Sanger Sequencing	circRNA identification and validation	100 ng	- Well-established technology - Cost-effective - Specific - Gold standard for circRNA validation	- It may require time to test divergent primers - Optimization is required for each pair of primers - Does not allow multiplexing	Panda *et al*.^[89]^, 2018
Northern Blot	circRNA identification and validation	1-50 µg	- Specific circRNA detection - Allows isoform studies - Solves those problems attained to qRT-PCR such as template switching or rolling amplification biases	- Low sensitivity - It requires a big amount of input which makes it incompatible with most liquid biopsy downstream processes	Schneider *et al*.^[128]^

The range of possibilities when selecting a bio-source is rather ample^[75]^. Whilst plasma or serum can provide a higher yield of total RNA, tumor released EVs stand out by providing a more accurate picture of lung cancer at the transcriptional level^[76]^. Procedures such as ultracentrifugation, ultrafiltration, or size-exclusion chromatography are examples of the range of methods accepted by the International Society for Extracellular Vesicles for the study and purification of these biomarkers^[77]^.

In the case of EV circRNA investigation, concentration levels may sometimes be the limitation factor that restricts further downstream processes. Therefore, in this case, EV isolation methods should be focused on achieving a higher EV-derived circRNA yield rather than acquiring extra pure EV samples, which are mainly attained by compromising RNA concentration^[78]^.

#### De novo discovery of circRNA

Full-length RNA sequencing emerged as the first method proving beneficial for *de novo* circRNA identification^[9]^. By processing total RNA, unmatched reads are selected and assembled by remapping to custom databases containing all human intragenic exon-exon junctions. This protocol first introduced by Salzman *et al*^[79]^. has since been improved with new procedures including ribosomal RNA depletion and non-polyadenylated RNA exonuclease-mediated enrichment (RNase R)^[79]^. Further validation of novel identified targets requires use of specific bioinformatic tools that allow junction site identification from deep-sequencing data. The rise of newly developed bioinformatic methods have boosted the discovery and analysis of thousands of circRNA [Table 5]. However, sensitivity may be a limitation when using next-generation sequencing for circRNA discovery since library preparation is frequently associated with the loss of low-expressed molecules^[80]^. Other methodologies such as microarrays or the nCounter platform have emerged to overcome this issue; however, circRNA discovery in these cases gets restricted to the candidates included either in the array or the gene panel.

**Table 5:** Characteristics of online accessible circRNA resources

**Name**	**Resource**	**Features**	**Website**	**Ref.**
circBase	Database	One of the main resources with updated information discovered circRNAs. Provides a useful blat tool for circRNA alignment against the human genome	http://www.circbase.org	Garcia-Contreras *et al*.^[84]^, 2014
circBank	Database	Along with circBase, is one of the most important resources available including a database with most discovered circRNAs along with usegul information	http://www.circbank.cn	Liu *et al*.^[93]^, 2019
circInteratome	Database	Complete database with different features that allow binding site prediction and knock-down experiment designing	https://circinteractome.nia.nih.gov	Dudekula *et al*.^[103]^, 2016
CIRCpedia	Database	Database for the identification of tissue specific circRNAs	http://www.picb.ac.cn/rnomics/circpedia	Dong *et al*.^[104]^, 2018
circRNADb	Database	Searching tool for the identification of EcircRNAs.	http://reprod.njmu.edu.cn/circrnadb	Chen *et al*.^[105]^, 2016
circRNABase	Database	Allows circRNA network prediction	http://www.hzrna.com/circrn-shujuku/circrnabase	circRNABase^[106]^, 2016
circR2Disease	Database	Serves for the identification of circRNA-miRNA interactions associated to different diseases	http://bioinfo.snnu.edu.cn/CircR2Disease/	Fan *et al*.^[107]^, 2018
starBase	Database	Serves for the identification of circRNA-miRNA interactions	http://starbase.sysu.edu.cn/	Li *et al*.^[108]^, 2014
circAtlas	Database	Databased with annotation of circRNAs and with tools that allow identification of circRNA-miRNA interactions	http://circatlas.bols.ac.cn/	Wu *et al*.^[109]^, 2020
circFunBase	Database	A database for functional circRNAs	http://bis.zju.edu.cn/CircFunBase	Meng *et al*.^[110]^, 2019
circad	Database	Serves for the identification of circRNA-miRNA interactions associated to different diseases	http://clingen.igib.res.in/circad/	Rophina *et al*.^[111]^, 2020
circView	Visualization tool	Identification circRNA associated miRNAs and RBPs	http://gb.whu.edu.cn/CircView/	Feng *et al*.^[95]^, 2018
CSCD	Bioinformatic tool	Identification circRNA associated miRNAs and RBPs, with a focus on circRNA with transcription potential	http://gb.whu.edu.cn/CSCD/	Xia *et al*.^[112]^, 2018
cirRNAPL	Bionformatic tool	Identification of circRNA based on extreme learning machine	http://server.malab.cn/CirRNAPL/index.html	Niu *et al*.^[113]^, 2020
nSolver	Program-Bioinformatic tool	Analysis of RNA expression data generated by the nCounter platform	www.nanostring.com	-
circ2Traits	Pipeline	Serves for the identification of circRNA-miRNA interactions associated to different diseases	http://gyanxetbeta.com/circdb/	Ghosal *et al*.^[114]^, 2013
circMeta	Pipeline	Genomic feature annotation and differential expression analysis of circular RNAs	https://github.com/lichenlab/circMeta	Chen *et al*.^[115]^, 2020
circRNAwrap	Pipeline	Pipeline designed for circRNA identification, transcript prediction, and abundance estimation	https://github.com/liaoscience/circRNAwrap	Li *et al*.^[116]^, 2019
SpliceV	Pipeline	Analysis and publication quality printing of linear and circular RNA splicing, expression and regulation	https://github.com/flemingtonlab/SpliceV	Ungerleider *et al*.^[117]^, 2019
CIRCexplorer3	Pipeline	Pipeline for the direct comparison of circular and linear RNA expression	https://github.com/YangLab/CLEAR	Ma *et al*.^[118]^, 2019
circDeep	Pipeline	Permits circular RNA classification from other long non-coding RNA	https://github.com/UofLBioinformatics/circDeep	Chaabane *et al*.^[119]^, 2020
Segemehl	Pipeline	Pipeline for the identification of fusion reads	http://www.bioinf.uni-leipzig.de/Software/segemehl/segemehl_0_2_0.tar.gz	Hoffmann *et al*.^[120]^, 2014
MapSplice	Pipeline	Application for small segment mapping	http://www.netlab.uky.edu/p/bioinfo/MapSpliceDownload	-
DCC	Pipeline	Identification of circRNA from fusion reads	https://github.com/dieterichlab/DCC	Cheng *et al*.^[121]^, 2016
UROBORUS	Pipeline	Allows identification of EcircRNAs	https://github.com/WGLab/uroborus/	Song *et al*.^[122]^, 2016
NCLscan	Pipeline	Identification of non-coding transcripts	https://github.com/TreesLab/NCLscan	Chuang *et al*.^[123]^, 2016
Trcirc	High-throughput Data analysis tool	Allows the prediction of circRNA-transcription factor regulatory networks	http://www.licpathway.net/TRCirc/	Tang *et al*.^[124]^, 2018

Microarrays are useful tools for high-throughput analysis and expression studies of circRNAs where probes are designed to bind specifically to the junction site, getting immobilized, incubated, and further sequenced^[81]^. Samples may normally be subject to RNase R to reduce background noise and enhance detection. This systematically expression profiling process is quite sensitive and straight forward. Current methodology developed by Arraystar includes all necessary tools in order to get detailed annotation specific to circRNA biology, such as miRNA binding sites or conservation status, to reveal all possible functional roles as miRNA sponges.

The nCounter platform allows multiplex analysis of up to 800 circRNA transcripts by direct capturing and counting of individual targets^[82]^. This qualitative and quantitative process is rather simple and requires minimal hands on, providing results in less than 48 h. Although nCounter is routinely used for RNA expression assessment in both FFPE and fresh tissues, only few studies have investigated its potential when it comes to liquid biopsies. EV-DNA^[83]^ and EV-miRNA^[84]^ profiles have been examined with this platform obtaining different success rates; however, investigation with circRNA remains restricted to tumor and cultured cells^[85]^, and in no case this platform has been explored for lung cancer research so far.

#### CircRNA identification and validation

For circRNA validation, end-point PCR has been established as the most extended practice using divergent primers spanning the junction site and followed by further Sanger sequencing^[63]^.

RNase R treatment is still a debate whether it is beneficial or not to use it in liquid biopsy samples. RNase R has been widely used for the study of circRNAs since it has the property of affecting mostly linear RNA, henceforth, enriching our samples with circRNAs^[86]^. However, some circRNAs have demonstrated to be sensitive to the effect of this exonuclease^[85]^. The often-long incubation periods can compromise the quality of our RNA samples. In addition, RNase treatment has been proved to not be 100% effective towards mRNA depletion which could lead to a circRNA overestimation if quantification by qPCR is the next downstream process and convergent primers are used. Xiao *et al*.^[87]^ proved that standard RNase R protocols result in up to 20% of highly expressed mRNAs being unaffected. Therefore, the correct design of divergent primers is instrumental for the study of circRNAs, regardless of whether RNase R treatment is applied to the samples or not. Authors also described that RNase R protocol could be enhanced by replacing K^+ ^by Li^+^ in the reaction buffer so enzyme can digest complex structured linear transcripts; however, this is a convoluted process that, even though scientifically relevant, may not result practical in the laboratory routine.

Northern blot analysis has arisen as another common methodology for the study of circRNAs. Following standard protocols, once the RNA is transferred from the gel onto a blotting membrane, circRNAs are then hybridized with short probes normally designed spanning the junction site, hence, allowing circRNA identification. This method also allows studies on size, isoforms, sequence, and abundance of these circular transcripts^[88]^. However, the usual high amounts of RNA required for this method is rather high, so investigations get restricted mostly to RNA from either tissue or cell lines.

#### Quantification of circRNA

Nowadays, different methodologies are being used for the quantification of circRNAs both in solid and liquid biopsies. qRT-PCR has been broadly established as one of the easiest and predilected mechanisms of quantification^[89]^; however, different aspects may need to be taken into consideration.

Contrary to tissue, circRNAs are enriched in plasma exosomes^[61]^. In this case, RNase R treatment may not be recommended due to the low overall RNA concentration that is expected in these vesicles, however, sometimes its use is necessary to validate primer specificity or due to the nature of specific experiments. In this respect, it is important to stress the need of designing divergent primers as previously cited, along with a probe spanning the junction site. Furthermore, throughout this procedure, the expression of classical reference genes, such as *beta-actin* or *GADPH*, will result altered; hence, ruling out the possibility of performing circRNA expression evaluation by using classical normalization procedures. In this case, the selection of circular RNA housekeeping genes^[90]^ is crucial for the correct assessment of circRNA expression.

CircRNA amplification via reverse transcription PCR (RT-PCR) often leads to extended concatemeric transcript amplification from a single priming of the reverse transcriptase. This process, triggered by the circular architecture of these molecules, is known as rolling circle amplification, and was first described by You *et al*.^[91]^ while studying circRNA expression in brain tissues. This event is not problematic if *de novo* circRNA discovery is intentional and direct comparison with canonical transcripts is not envisioned (in fact, it can be beneficial for the study of circRNA splice variants). However, this does not apply to transcript abundant studies, in which this mechanism can introduce biases leading to an overestimation of circRNA expression.

Conn *et al*.^[92]^ demonstrate this in a study with synthetic circRNAs, resulting in a five-fold increase of circRNAs compared to the expected expression upon RT-PCR and further qPCR amplification. This is a factor to take into consideration in the experimental design^[92]^.

The same group has developed a cutting-edge tool to avoid the bias introduced by normal qRT-PCR quantification throughout their newly designed SplintQuant method^[92]^. This technology is based on the inclusion of custom DNA oligonucleotides that complement target circRNAs, and making use of the PBCV-1 DNA ligase, synthesize cDNA skipping reverse transcription. The system is sensitive, specific and reproducible, allowing the identification and quantification of canonical and non-canonical RNA transcripts including gene fusions and alternative splice variants.

nCounter technology stands out as a very effective and sensitive option for circRNA quantification. Its application for the analysis and quantification of circRNAs has been systematically studied by Dahl *et al*.^[85]^ in different solid biosources (including formalin fixed paraffin-embedded specimens) for the study of B-cell malignancies, becoming the first group to use this technology for the study of circRNA expression.

#### Bioinformatic and computational tools for the study of circRNA

Identification of circRNAs can be a straight-forward process when using microarray or nCounter data where the exploratory approach gets restricted to a specific panel of genes. However, detection of circRNA can be a much more complex in the case of deep-sequencing data analysis due to the complexity on the computational workflows. For this purpose, different pipelines and computational analysis tools have been created to facilitate this process [Table 5]. Different publicly available databases such as circBank^[93]^, circBase^[94]^, or circView^[95]^ have proved useful to simplify the study of circRNA throwing light on specific features such as miRNA binding sites, m6A modifications, mutations, or unveiling protein-coding potential [Table 5]. These databases also allow browsing and download of FASTA files based on specific searching criteria.

## DISCUSSION

The recent impact of circRNAs in lung cancer research has become undeniable. Since ciRS-7 was introduced as the first circRNA ever described to play a role in hepatocellular carcinoma^[36]^, many others have followed, extending to different types of cancer, henceforth, consolidating their position as active players in cancer development and progression of malignancy. Recently, publications exploring the biomarker potential of these molecules in NSCLC have remarkably increased, with an exponential growth in the last five years. Nevertheless, despite the patent progress in this field, current research is predominantly restricted to expression analysis of circRNA in tumor samples, with very little information regarding validation in liquid specimens.

EVs, including exosomes, are released by most cells in the body and can be easily isolated from plasma^[96]^. Tumor EVs can mediate intercellular communication between tumor cells and tumor microenvironment^[97]^; therefore, the study of these molecules via their molecular identification can offer a valuable spatiotemporal snapshot of the state of the disease. However, while several publications have widely demonstrated that EV cargo is enriched in circRNAs^[61]^, not many investigators have focused on this line of research, delaying the development of novel liquid biopsy-based tools for NSCLC detection. While the potential value of liquid biopsies in the clinic has been recognized as beneficial^[98]^, in the research context, liquid bio-sources can be rather challenging, including plasma circRNA investigation.

With a superior relative expression and stability in EVs than the canonical mRNA, the extent of circRNA in EVs still remains very low, frequently limiting further downstream analysis. This is unlikely to be an issue in solid tumors; while circRNA overall expression is frequently low (1%-10%)^[14]^, RNA concentration is rarely a limitation. Furthermore, very often the study of circRNA expression relies on enzymatic amplification - qPCR. This course fueled by the circular architecture of these molecules can sometimes lead to the not-so-well-known rolling cycle amplification events, resulting in an inaccurate yet overestimated circRNA quantification^[92]^, frequently leading to untruthful and irreproducible results.

On addition to the above exposed, there is not a general consensus about other fundamental matters such as EV isolation method (if we target the study of the EV circRNA cargo), potential use of RNase R, or readout assessment, among others. As a result, standardization of protocols for the study of circRNA has become instrumental for the study and implementation of these novel biomarkers into the liquid biopsy setting.

Some technologies have arisen as incipient alternatives such as the nCounter platform or the newly developed SplintQuant. Both of them rely on very low RNA input and can overcome the deviation issues that enzymatic qPCR may create.

Additionally, platelets, especially tumor educated platelets, hold a great unexplored potential as a source of circRNAs, not only due to their higher concentration in RNA when compared to EVs, but also due to the high enrichment they present towards these circular biomolecules. To elucidate wheater platelet derived circRNA signatures could be of better, equal, or complementary value of the ones from EVs, additional investigation will be required.

Nowadays, most studies aim to exploit the biomarker potential of lung cancer circRNAs, frequently leaving aside any additional examination of their inherent biology. Further research elucidating the different molecular functions of these molecules is greatly needed in order to achieve a future circRNA-based liquid biopsy test.

The rediscovered role of circRNAs as lung cancer biomarkers has the potential to reshape the landscape of liquid biopsies. They count on most features needed to be considered a good biomarker: they can be measured in blood^[99]^, including plasma^[68]^, serum^[100]^, and urine^[101]^; they are reasonably robust and very stable due to their circular architecture^[34]^; and do not require special handling protocols other than those required for the rest of RNA types. Due to the diverse implications in cancer progression and development of resistance^[34]^, circRNAs could provide additional information improving diagnosis and treatment guidance by either generating new signatures, or complimenting existing ones.

Circulating tumor DNA is the most commonly explored liquid biopsy for NSCLC, counting with few tests already clinically implemented for the detection of classical mutations such as *EGFR Del19* and *p. L858R* mutation^[102]^. However, many lung cancer cases are not linked to a specific driver mutation; therefore, research on new biomarkers, including circRNAs, and further development of multi-omic signatures of tumor microenvironment could provide additional diagnostic opportunities for these patients.

However, as mentioned above, several circRNA quantification methods have limitations, and a clear protocol needs first to be established in order to develop any clinically applicable assay. In addition, clinical utility should be demonstrated by providing convincing evidence of the new biomarker performance (in comparison to currently accepted cfDNA/mRNA liquid biopsy tests), and so far, no circRNA biomarker has achieved that status, probably due to the difficulty of recruiting large patient cohorts required to prove biomarker utility.

Further studies in biomarker discovery, molecular biology, and protocol standardization are warranted in the upcoming years to achieve the implementation of these novel biomarkers in the clinical setting.

## DECLARATIONS

### Copyright

© The Author(s) 2021.
